# Endoscopic ultrasound-guided hepaticojejunostomy through an endoscopic ultrasound-guided enteroanastomosis

**DOI:** 10.1055/a-2548-6760

**Published:** 2025-03-12

**Authors:** Luis Araya-Acero, Maria Lynch-Mejia, Fernanda Castro-Gonzalez, Jorge Vargas-Madrigal

**Affiliations:** 1 27915 Department of Gastroenterology, University of Costa Rica, San José, Costa Rica; 2 42697 Department of Gastroenterology, Caja Costarricense de Seguro Social, San José, Costa Rica

Endoscopic retrograde cholangiopancreatography (ERCP) in surgically altered anatomy is a challenging procedure 1 . Transjejunal ERCP through endoscopic ultrasound (EUS)-guided enteroanastomosis appears to be a safe and effective technique 2 . We report a 65-year-old woman with a previous Roux-en-Y hepaticojejunal anastomosis presenting with acute bacterial cholangitis complicated by septic shock. She underwent unsuccessful enteroscopy-assisted ERCP. Given her altered anatomy, an EUS-guided approach was offered ( Video 1 ).

Endoscopic ultrasound-guided hepaticojejunostomy through an endoscopic ultrasound-guided enteroanastomosis.Video 1

A forward-viewing scope was advanced to the afferent limb, and a nasobiliary drainage (G21583; Cook Medical, Bloomington, Indiana, USA) was placed with connection to a water pump. A linear echoendoscope (GF-UCT 180; Olympus, Tokyo, Japan) connected to an ultrasound processor (Arietta 850; Fujifilm, Tokyo, Japan) was used, and by pumping water into the afferent limb this provided a clear view for creating a duodenojejunostomy with a 15-mm lumen-apposing metal stent (LAMS) (Axios; Boston Scientific, Marlborough, Massachusetts, USA) to access the biliary tree ( Fig. 1 ). After allowing time for tract maturation, the duodenojejunostomy was accessed with a duodenoscope, but cannulation was not possible due to a very tight angulated stricture. A decision was made to access the LAMS with a forward-viewing echoendoscope (TGF-UC180J; Olympus) to create a new biliary anastomosis. The common hepatic duct was identified and punctured with a 19G needle ( Fig. 2 ) (EZ Shot 3 Plus; Olympus). Confirming adequate location with a cholangiogram ( Fig. 3 ), a 0.025-inch guidewire (VisiGlide; Olympus) was advanced. A cystotome (G30550; Cook Medical) was fed in, and a new hepaticojejunostomy was created and dilated up to 12 mm with a pneumatic balloon ( Fig. 4 ) (CRE Rx; Boston Scientific). To finalize, 3 biodegradable 10 Fr stents (Archimedes; Q3 Medical Group, Dublin, Ireland) were placed. After successful drainage the patient improved, and no complications related to the procedure were documented.

**Fig. 1:**
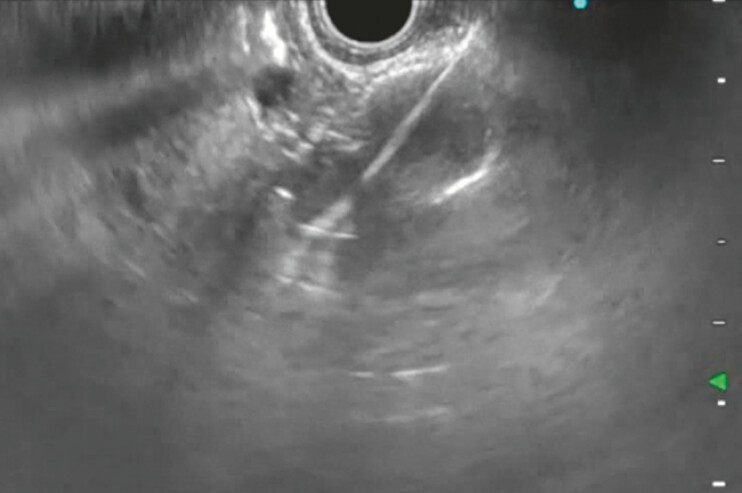
Ultrasound image showing placement of lumen-apposing stent to access the biliary tree in a 65-year-old woman with acute bacterial cholangitis and altered anatomy after previous Roux-en-Y hepaticojejunal anastomosis.

**Fig. 2:**
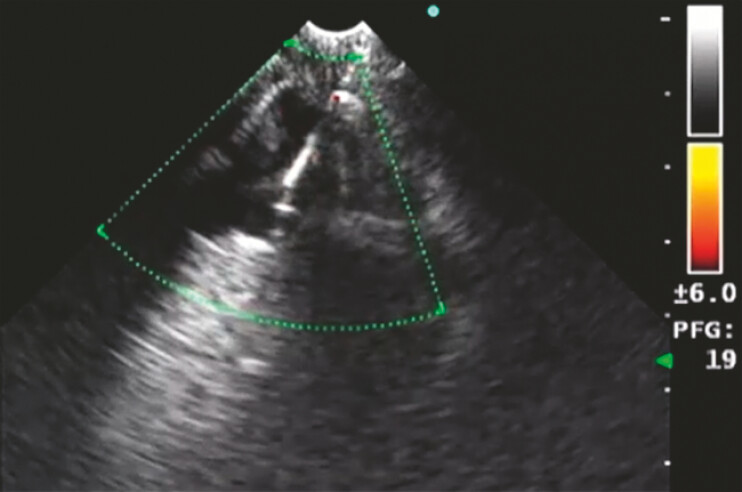
Ultrasound image showing common hepatic duct punctured by a 19G fine-needle aspiration needle.

**Fig. 3:**
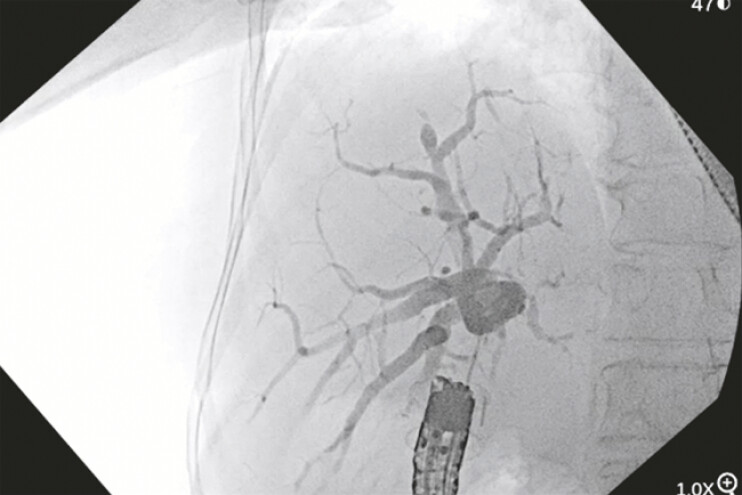
Endoscopic ultrasound (EUS)-guided cholangiogram showing successful puncture of the common hepatic duct.

**Fig. 4:**
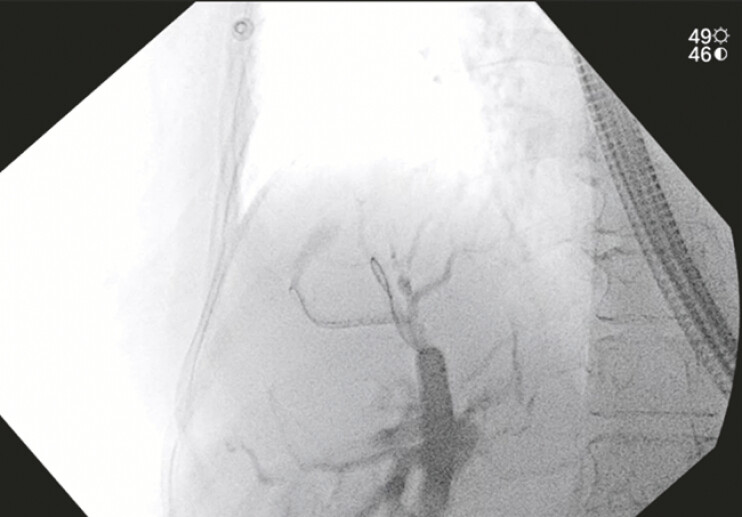
Cholangiogram showing balloon dilation of the newly created EUS-guided hepaticojejunostomy.

EUS-guided enterostomy with hepaticojejunostomy drainage offers a novel alternative for bile duct access in patients with altered anatomy.

Endoscopy_UCTN_Code_TTT_1AS_2AH

## Notes

Endoscopy E-Videos https://eref.thieme.de/e-videos
*E-Videos* is an open access online section of the journal *Endoscopy* , reporting on interesting cases and new techniques in gastroenterological endoscopy. All papers include a high-quality video and are published with a Creative Commons CC-BY license. Endoscopy E-Videos qualify for HINARI discounts and waivers and eligibility is automatically checked during the submission process. We grant 100% waivers to articles whose corresponding authors are based in Group A countries and 50% waivers to those who are based in Group B countries as classified by Research4Life (see: https://www.research4life.org/access/eligibility/ ). This section has its own submission website at https://mc.manuscriptcentral.com/e-videos .
